# Genetics of range expansion and admixture of *Aedes aegypti* populations in California

**DOI:** 10.1186/s12864-025-12443-7

**Published:** 2025-12-19

**Authors:** Melina Campos, Yoosook Lee, Katherine Brisco, Marc Crepeau, Anthony J. Cornel, Gregory C. Lanzaro

**Affiliations:** 1 Department of Pathology, Microbiology and Immunology, Vector Genetics Laboratory, UC Davis, Davis, CA USA; 2 Department of Entomology and Nematology, Florida Medical Entomology Laboratory, Institute of Food and Agricultural Sciences, University of Florida, Vero Beach, FL USA; 3 Department of Entomology and Nematology, Mosquito Control Research Laboratory, University of California, Parlier, CA USA

**Keywords:** Hybridization, Invasive species, Human-mediated dispersal, Mosquito, Vector borne diseases, Arbovirus

## Abstract

**Background:**

The mosquito *Aedes aegypti*, a key vector for arboviruses including dengue, Zika, and chikungunya, was first detected in California in 2013 and has since expanded northward. This study examines the genetic structure of California populations and, based on that structure, proposes potential mechanisms driving their invasion across the state.

**Results:**

A whole-genome analysis of 181 individuals, including 49 newly sequenced from recently established populations in Northern California, corroborates previously described genetic structure and reveals the origins of these populations. Many northern populations shared ancestry with Southern California populations, suggesting passive dispersal. Additionally, we observed significant genetic admixture between divergent clusters in the Central Valley, associated with increased nucleotide diversity, which may enhance adaptive potential. We describe the effects of range expansion and genetic admixture on divergent ancestral lineages and discuss the importance of human-mediated dispersal in the spread of this invasive species.

**Conclusions:**

Our results illustrate the utility of genomic tools in surveillance programs for tracking dispersal patterns. Such strategies can contribute to mitigating the growing public health threat posed by *Ae. aegypti*'s continued expansion in California, particularly as locally acquired arbovirus cases increase.

**Supplementary Information:**

The online version contains supplementary material available at 10.1186/s12864-025-12443-7.

## Background

Introduced species commonly experience population bottlenecks upon founding. The consequent reduction in genetic diversity, coupled with exposure to a novel, potentially challenging environment, may threaten their survival. In fact, the majority of introduced species fail to become established [1]. Some invasive species are capable of overcoming these barriers to become successfully established in a new environment [2]. However, reduced genetic diversity in initial founding populations may limit their invasive potential. Mechanisms that facilitate an increase in genetic diversity may serve to increase a population’s ability to adapt and spread. One such mechanism is hybridization among sub-populations [3]. Species invasions frequently involve multiple introductions resulting in genetically distinct sub-populations [4]. If range expansion brings sub-populations into contact, and hybridization and introgression are likely to occur. This may increase fitness by heterosis or by the formation of novel genotypes that are adaptive [5].

In addition to multiple introductions, invasions over broader geographic space may be facilitated by the spread of individuals from initial, well-established populations, the so-called bridgehead effect [6]. Widespread invasions sourced from a bridgehead population may be facilitated by human-mediated dispersal (HMD), where individuals are transported, potentially over significant distances by human trade and travel [7, 8]. In this paper we describe the results of a genomics-based analysis of established populations of the mosquito, *Aedes aegypti* in California and how this mosquito is moving and potentially adapting to this new environment.

*Aedes aegypti* is native to Africa but has spread globally to subtropical and tropical areas on every continent except Antarctica [9, 10]. This expansion is believed to have been initiated in the sixteenth century through transatlantic voyages associated with the slave trade, during which *Ae. aegypti* eggs and larvae were likely transported in water supplies on ships [11, 12]. Numerous studies have highlighted this mosquito’s weak flight capability but strong capacity to “hitchhike” via HMD, enabling it to travel considerable distances over short periods [13–17]. In the case of *Ae. aegypti*, HMD likely occurs via transport of desiccation-resistant eggs which are frequently deposited in artificial containers, such as water storage vessels and other human-associated items, which are easily transported via ship and ground transportation [18, 19].

In the summer of 2013, *Ae. aegypti* was first detected in California, with initial appearances in three Central California cities: Clovis, Madera, and Menlo Park [20, 21]. Although earlier introductions may have occurred, these populations likely remained undetected or failed to persist, given the historically intensive mosquito surveillance in the state [22, 23]. Since then, this species has become established and expanded its range across the Central Valley and into Northern California (Supplementary Fig. 1). The continued spread of *Ae. aegypti* poses a significant public health threat due to its capacity to transmit arboviruses including dengue, Zika, and chikungunya viruses. There have been no autochthonous cases of either chikungunya or Zika in California, but locally acquired dengue infections have been documented in the Greater Los Angeles and San Diego areas in 2023 and 2024 [20].

Three genetically distinct populations of *Ae. aegypti* have been described in California, presumably representing three independent invasions [23, 24]. Different populations are established in coastal and central Southern California (GC1) and the Central Valley (GC2 and GC3). This study aims to explore the spatial and temporal dynamics driving *Ae. aegypti*’s successful range expansion within the state. While some Northern California populations may have arisen through gradual migration via active dispersal from nearby Central California populations, the possibility of passive dispersal from more distant locations cannot be excluded. For example, a recent study on the reintroduction of *Ae. aegypti* in Exeter, Central California, after a successful elimination program, revealed that the source population originated from Southern California rather than nearby sites [25].

More specifically, in this paper we used whole-genome sequencing of 182 individual *Ae. aegypti* specimens to investigate the origins of recently introduced populations of the mosquito in California. These samples were collected from across the state at various time points to describe both the spatial and temporal genetic structure of the populations. Using this information, we assessed genetic differentiation among subpopulations and changes in their range. We identified instances where the range of genetically distinct subpopulations have expanded and now overlaps. We describe how individuals from these subpopulations interact when in sympatry. We also investigated the origin of spatially disjunct and recently identified populations.

Populations of *Ae. aegypti* were introduced into California only 12 years ago and so provide a unique opportunity to observe rapid evolution as this tropical species adapts to the vast array of habitats within the central valley of California. From a practical standpoint, the results presented here may inform strategies aimed at controlling this highly invasive disease vector species.

## Methods

### Sampling

Various Mosquito Abatement Districts (MADs) assisted in the collection of adult female *Ae. aegypti* from seven sites across California: Chico, Citrus Heights, Sacramento, Shasta, Stockton, Winters and Yuba (Supplementary Table 1). Collections were conducted by MAD technical personnel using BG-sentinel traps baited with CO_2_ or other lures [26]. Each individual mosquito sample was preserved in a 0.5 ml tube containing 80% ethanol before DNA extraction. Complementary genome data from 47 different locations in California were obtained from publicly available sequences (Bioproject: PRJNA385349; Supplementary Table 1).

### Whole genome sequencing

DNA extraction from individual mosquitoes was performed using the Qiagen BioSprint kit, following an established protocol by Nieman et al. [27]. DNA quantification was carried out using the Qubit dsDNA HS Assay Kit and a Qubit fluorometer (Thermo Fisher Scientific, Waltham, MA, USA). Genomic DNA libraries were prepared using 10 ng of DNA input per sample, employing the KAPA HyperPlus Kit (Roche Sequencing Solutions, Indianapolis, IN, USA), as detailed by Yamasaki et al. [28]. Library size selection and purification were conducted using AMPure SPRI beads (Beckman Coulter Life Sciences, Indianapolis, IN, USA). Libraries were sequenced as 150 bp paired-end reads on a HiSeq 4000 instrument (Illumina, San Diego, CA, USA) at the UC Davis DNA Technologies Core Facility.

### Sequencing data processing

Demultiplexed raw reads were quality-filtered and trimmed using Trimmomatic v0.36 [29]. Reads were mapped to the *Ae. aegypti* AaegL5 reference genome [30] using BWA-MEM v0.7.15 [31]. The mapping process followed the recommendations of Schmidt et al. [32], where reads were first aligned to the mitochondrial reference genome. Unmapped reads were subsequently mapped to the nuclear genome. Mapping statistics were calculated with Qualimap v2.2 [33]. Joint variant calling across all samples was performed using FreeBayes v1.0.1 [34] with standard filters and population priors disabled. Only biallelic SNPs meeting the following criteria were retained for analysis: minimum quality score of 20, minimum depth of 8 reads, no more than 5% missing data across the dataset, and minimum allele frequency (MAF) of 5%. For sensitivity comparisons, selected analyses were repeated without MAF filtering to evaluate whether inclusion of rare variants altered the overall patterns.

### Population structure

Linkage disequilibrium (LD) pruning was conducted using scikit-allel v1.3.0 [35] with a window size of 50 SNPs, a step size of 5 SNPs, and a threshold of 0.1. A subset of 50,000 SNPs was selected for Principal Component Analysis (PCA), which was performed and visualized in R [36]. Pairwise fixation indices *F*_*ST*_ were calculated using Hudson's estimator [37], as implemented in scikit-allel [35]. A neighbor-joining tree was constructed based on pairwise *F*_*ST*_ values using the ape package in R [38].

Population structure was further investigated by assigning individual genomes to genetic clusters using ADMIXTURE v1.3 [39]. Three independent replicates of 50,000 SNPs each were submitted for admixture analysis. For each replicate, ten iterations were run with *K* values ranging from 1 to 15. Results were compiled using CLUMPAK [40], and the optimal *K* value was determined based on the lowest cross-validation error.

### Genetic clusters admixture

To confirm the intermediate genetic ancestry between GC2 and GC3, a pairwise genomic scan was conducted with mean *F*_*ST*_ values calculated in non-overlapping 100 kb windows. Ancestry informative markers (AIMs) were identified as SNPs with *F*_*ST*_ values greater than 0.95 between GC2 and GC3. The percentage of ancestry for each sample was calculated based on these AIMs. Nucleotide diversity (π) was calculated in non-overlapping 100 kb windows using VCFtools [41]. Heterozygosity was calculated on a per-individual basis using VCFtools [41].

## Results

### State-wide population structure

**Fig. 1:**
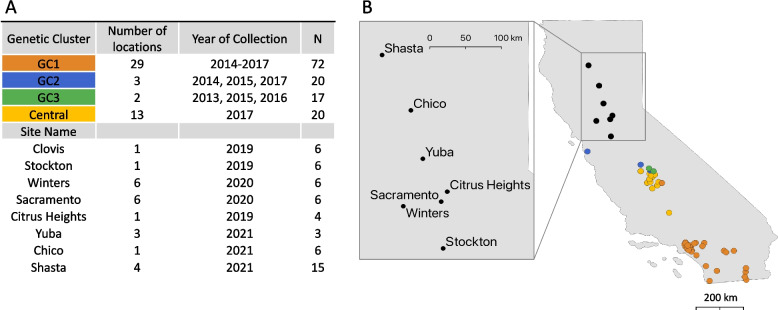
Study sampling locations. Geographic locations of sampled *Aedes aegypti* populations, with colors corresponding to previously identified genetic clusters: GC1, GC2 and GC3. **A**) Sample size for each genetic cluster (GC) or site and year of collection. **B**) Map of California displaying the sampling locations. Inset provide detailed views of newly invaded locations in northern California

A total of 49 new *Ae. aegypti* genomes were sequenced for this study and these were combined with an existing dataset of 132 genomes for analysis [24]. The newly sequenced genomes originated from several Northern California cities that were recently invaded (between 2019–2021), including Stockton (*N* = 6), Sacramento (*N* = 6), Citrus Heights (*N* = 4), Winters (*N* = 6), Yuba (*N* = 3), Chico (*N* = 6), and 4 localities in Shasta County: Buckeye (*N* = 4), El Reno (*N* = 4), El Rio (*N* = 4), and Enchanted (*N* = 3) (Fig. 1, Supplementary Fig. 2). Across all samples, the mean coverage depth was approximately 10.6 × (Supplementary Table 1). For consistency, we adopted the established nomenclature in Lee et al. (2019) [23] to describe the major genetic clusters (GCs) identified among *Ae. aegypti* populations in California. GC1 encompassed populations from 30 localities in Southern California, plus Exeter in the Central Valley; GC2 included nine populations predominantly from the Central Valley, and GC3 represented populations from Clovis (collected in 2013 and 2016) and Sanger.

**Fig. 2:**
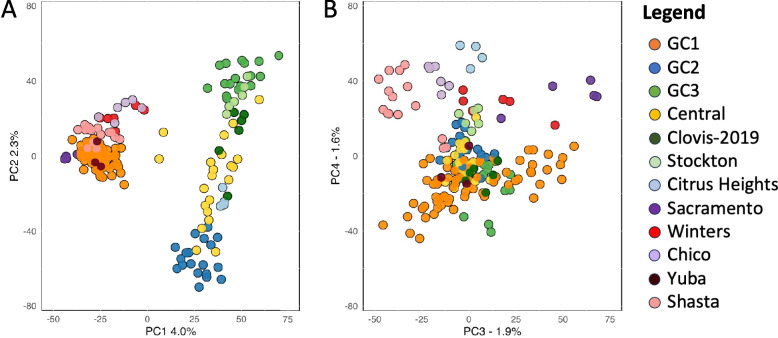
PCA analysis of genome wide SNPs of *Aedes aegypti*. Plot of the first four components of PCA, (**A**) PC1 and PC2. Colors highlight previously identified genetic clusters: GC1 (orange), GC2 (blue), and GC3 (green); new localities in the Californian Central Valley (yellow), and newly invaded northern regions (yellow). (**B**) PC3 and PC4. Newly established populations in the northern end of the Central Valley form distinct clusters (Shasta, Chico, Citrus Heights). All analyses were based on 50,000 SNPs across the genome

Principal component analysis (PCA) corroborated the genetic structure observed in previous studies, distinguishing the three major genetic clusters along the first two principal components (PCs) (Fig. 2A). Six Central Valley populations occupied an intermediate genetic space between GC2 and GC3, as did specimens collected in 2019 from Clovis and individuals from Citrus Heights (Fig. 2A). The PCA also revealed that the recently discovered Northern California populations are genetically closer to GC1, a genetic cluster predominantly from Southern California. PCs 3 and 4 (Fig. 2B) clearly distinguish recently established populations collected from the cities of Shasta, Chico, Sacramento, and Winters in the northern end of the Central Valley (Fig. 1).

**Fig. 3:**
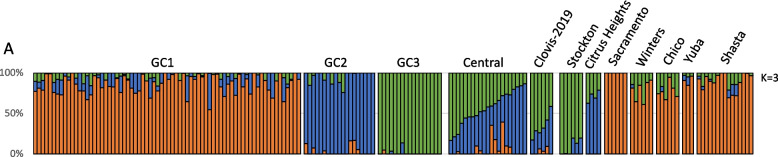
Bayesian analysis for ancestry estimation of *Aedes aegypti* populations in California. Analyses were based on 50,000 SNPs across the genome. Colors highlight previously described genetic clusters: GC1 (orange), GC2 (blue), and GC3 (green); new localities in the Californian Central Valley, and newly invaded northern regions

Admixture analysis supported the PCA results. When *K* = 3 (the optimal number of genetic clusters based on cross-validation error; Supplementary Fig. 3), the ancestry of individuals was primarily partitioned among GC1, GC2, and GC3 (Fig. 3). Populations from the Central Valley displayed varying degrees of admixture between GC2 and GC3, mirroring the PCA findings. Similarly, individuals from Citrus Heights and Clovis collected in 2019 (Fig. 1) showed admixture of GC2 and GC3 (Fig. 3). Stockton individuals predominantly shared genetic ancestry with GC3, while all other Northern California populations clustered with GC1 (Fig. 3). Results for alternative values of K (K = 4–6) are presented in Supplementary Fig. 4 and show consistent overall clustering patterns, with additional substructure within the newly invaded populations.

**Fig. 4:**
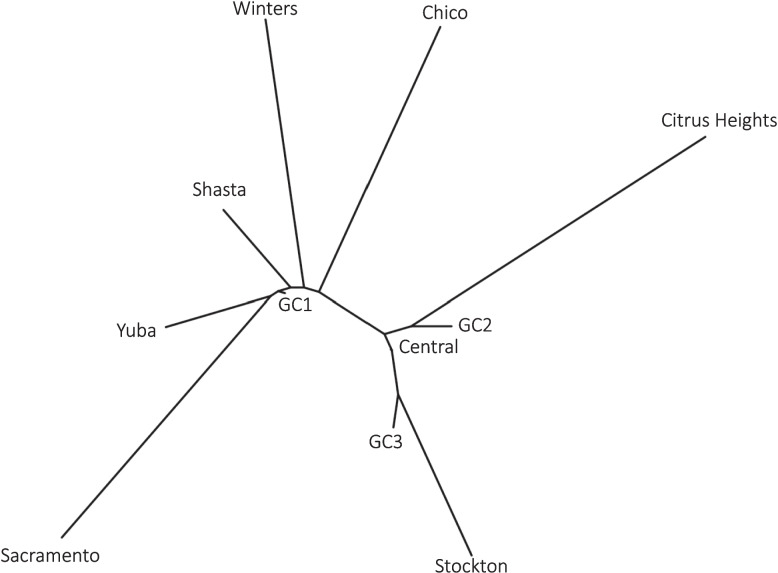
Neighbor joining tree of pairwise *F*_*ST*_ values. Pairwise *F*_*ST*_ between the newly invaded northern location in California and previously defined genetic clusters of *Aedes aegypti*

A neighbor-joining tree based on pairwise *F*_*ST*_ values was constructed solely as an exploratory visualization to summarize genetic relationships among the three major clusters and newly sampled northern populations (Fig. 4; Supplementary Table 2), rather than as a formal phylogenetic inference. (Fig. 4; Supplementary Table 2). This analysis showed that populations from Sacramento, Yuba, Winters, Shasta, and Chico were genetically closer to GC1. By contrast, Stockton aligned more closely with GC3, while Citrus Heights was genetically aligned with GC2. Analyses performed the MAF threshold produced consistent relationships (Supplementary Table 3).

### Genetic cluster admixture

**Fig. 5:**
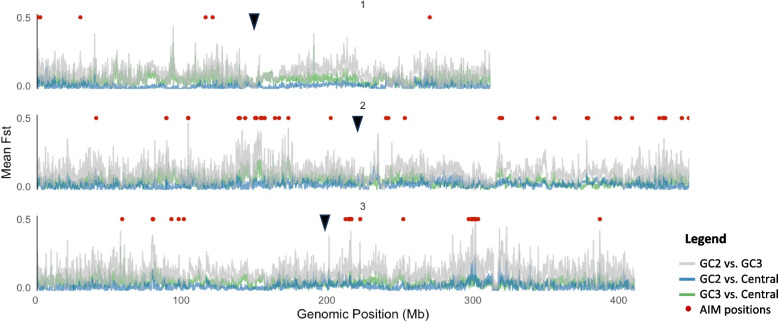
Genome scan comparison between pairs of genetic clusters. Non-overlapping sliding window *F*_*ST*_ between GC2 and GC3 in gray; GC2 versus Central in blue; and GC3 versus Central in green. Red dots indicate SNP positions of ancestry informative markers (AIMs) between GC2 and GC3; black arrows indicate the location of centromeres

To further investigate hybridization between clusters GC2 and GC3, we employed ancestry informative markers (AIMs). AIMs are biallelic single nucleotide polymorphisms (SNPs) that exhibit near-fixation within a specific genetic cluster, characterized by an *F*_*ST*_ value of 0.95 or higher. After filtering out SNPs located within 1,000 base pairs of one another to reduce linkage effects and ensure independence among markers, a total of 150 AIMs distributed across the genome were identified (Fig. 5; Supplementary Table 4). The mean *F*_*ST*_ between GC2 and GC3 was, as expected, higher (mean *F*_*ST*_ = 0.0981, p < 0.0001) than that for hybridized Central Valley populations versus GC2 (0.0102) and GC3 (0.0372). Windowed *F*_*ST*_ analyses across the genome confirmed this pattern. Genetic divergence (*F*_*ST*_) is consistently higher between GC2 and GC3 (Fig. 5).

**Fig. 6:**
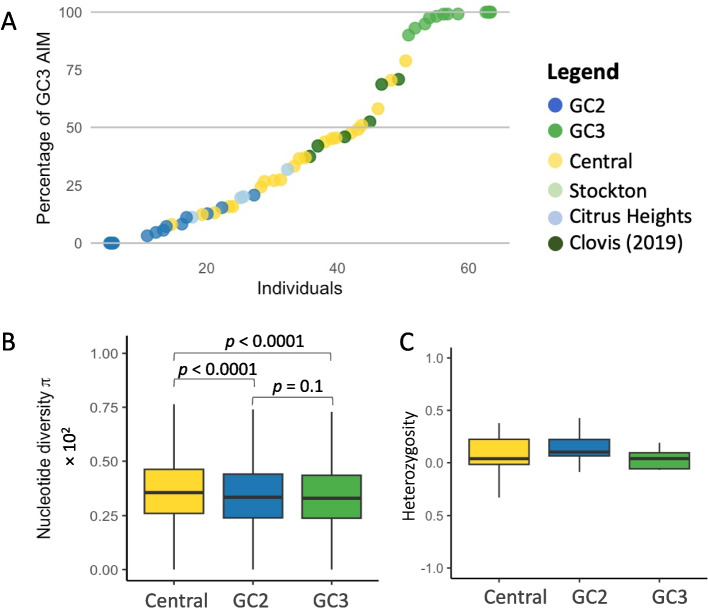
Population genetic statistics of two *Aedes aegypti* genetic clusters and admixture population. **A** Ranked scatter plot displaying the percentage of GC3 AIM in GC2, GC3, and new collections in Central Valley, Stockton, and Citrus Heights. **B** Boxplot of nucleotide diversity values calculated for non-overlapping 100,000 bp windows. **C** Boxplot of heterozygosity per-individual for each population

Populations from the Central Valley demonstrated mixed genetic ancestry, with contributions from GC3 ranging from approximately 8% to 79%, where 100% represents complete GC3 ancestry (Fig. 6A). Notably, individuals from Citrus Heights and Clovis (2019 collection) displayed similar intermediate patterns. AIM-based genetic ancestry in Citrus Heights ranged from 11 to 32%, while Clovis specimens ranged from 37 to 71% GC3 ancestry (Fig. 6A). Mean nucleotide diversity across the genome was significantly higher in mixed Central Valley populations compared to the genetic clusters GC2 and GC3 (Fig. 6B). The same qualitative pattern was observed when π was recalculated without MAF filtering (Supplementary Fig. 6). This elevated diversity likely reflects the hybridization and admixture between these two clusters, contributing to the genetic heterogeneity observed in hybrid populations. No clear pattern of heterozygosity was observed within populations, apart from a broader range of values in the mixed Central Valley population (Fig. 6C). The small sample size did not allow for statistical comparisons.

## Discussion

We provide new, updated insights into the genetic structure, range expansion, and admixture of *Ae. aegypti* populations in California. By integrating whole-genome sequencing data from Northern California with previously analyzed genomes, we confirmed and expanded upon existing knowledge of this mosquito's current population structure and dispersal patterns across the state. Our findings provide a new example of invasive species biology and support the development of new hypotheses regarding potential routes of *Ae. aegypti* establishment and spread in California. In addition, the genomic patterns we identify can be further explored to investigate genetic mechanisms that may facilitate adaptation in newly invaded environments. These results may have significant implications for vector control and public health management in the state.

The clear differentiation between the three major genetic clusters (GC1, GC2, and GC3) supports the hypothesis that *Ae. aegypti* populations in California originated from multiple introduction events. Consistent with prior studies [23, 24], GC1 represents the Southern California populations, while GC2 and GC3 are primarily located in the Central Valley. Our PCA and ADMIXTURE analyses reveal that more recent established populations in Northern California largely share genetic ancestry with GC1, suggesting that the spread into northern regions likely occurred via northward expansion from Southern California populations, likely by HMD.

A key finding of this study is the detection of admixture between GC2 and GC3 in Central Valley populations. Nonetheless, we acknowledge these methodological limitations, and we note that future work incorporating phylogenetic reconstruction and demographic modeling (supported by a validated outgroup and expanded reference populations) would provide valuable additional insight. This admixture likely results from overlapping range expansions and the persistence of both genetic clusters within close geographic areas. Elevated nucleotide diversity observed in these hybrid populations suggests that admixture could potentially increase the pool of genetic variation available to *Ae. aegypti*. Such diversity may provide a broader set of alleles for natural selection to act upon, which in theory could aid the species' ability to exploit diverse habitats and resist environmental stressors [42, 43]. However, we note that our study did not directly assess fitness or selection, so any implications for adaptive potential remain tentative. Patterns of heterozygosity did not differ markedly among populations, except for a wider range observed in the mixed Central Valley group. Genome-wide heterozygosity can be strongly influenced by inbreeding and demographic history, including recent bottlenecks or expansions, which may obscure underlying genetic patterns [44].

Interestingly, exceptions to the trend of northward expansion from Southern California were observed in Citrus Heights and Stockton, where individuals displayed intermediate genetic ancestries. The presence of admixed genetic ancestry in these northern populations indicates that the Central Valley may serve as a channel for gene flow between established clusters. This highlights the dynamic and ongoing nature of *Ae. aegypti*'s range expansion, driven by both active dispersal and HMD, with hybrid populations playing a key role in this process [45, 46].

The continued expansion of *Ae. aegypti* in California is a growing public health concern due to its ability to transmit arboviruses such as dengue, Zika, and chikungunya. Recent cases of locally acquired dengue in Los Angeles and San Diego [20] emphasize the potential for further outbreaks as the mosquito's range continues to expand and population densities increase. Therefore, monitoring genetic changes in *Ae. aegypti* populations over time may be useful for the detection of new introductions, tracking spread from bridgehead populations, and assessing spread due to travel and commerce which may contribute to the effectiveness of control programs using vehicle disinfection.

Whole-genome sequencing, as demonstrated in this study, is a powerful tool for uncovering impactful insights on *Ae. aegypti* dispersal in California. A limitation of the present work is that some populations were represented by small sample sizes, which limits the strength of population-level inferences for those localities. Building upon this foundation, less costly methodologies can be developed to create a small set of genetic markers to detect genetic clusters. A combination of genomics followed by expanded sampling using genotyping methods could facilitate more widespread and routine monitoring, supporting timely and region-specific vector control.

## Conclusions

This study provides insights into the mechanisms of dispersal and adaptation of *Ae. aegypti* populations in California, focusing on the northern expansion of the species. The results highlight a variety of dispersal mechanisms, including passive movement across large distances via HMD, which may be compounded by incremental active northward expansion from bridgehead populations in central regions. The detection of admixture between genetic clusters in the Central Valley illustrates the dynamic nature of *Ae. aegypti*’s range expansion, with hybrid populations potentially contributing to the species' adaptation to new environments. These findings suggest the utility of ongoing genetic surveillance and monitoring to track *Ae. aegypti*’s expansion in California, enabling timely interventions to mitigate the public health risks posed by this invasive vector.

## Supplementary Information

Supplementary Figure 1. Range expansion of *Aedes aegypti* in California. Map showing the distribution of positive traps for Ae. aegypti, aggregated by year from the initial species introduction in 2013 through 2023. Data provided by the California Department of Public Health.

Supplementary Figure 2. Additional map with all sampling location names. Sampling locations of Aedes aegypti populations in California with location names. Colors correspond to previously identified genetic clusters: GC1 (orange), GC2 (blue), and GC3 (green). Insets provide detailed views of sampling sites in the Central Valley and Northern California.

Supplementary Figure 3. Cross-validation error for K from 1 to 15 of ADMIXTURE analysis; the lowest value is the best-fit number of clusters.

Supplementary Figure 4. Bayesian analysis for ancestry estimation of Aedes aegypti populations in California. ADMIXTURE analysis of Aedes aegypti populations in California at alternative numbers of genetic clusters (K = 4, 5, and 6). Analyses were based on 50,000 SNPs across the genome. Colors represent genetic ancestry components.

Supplementary Figure 5. Nucleotide diversity across the genome of Aedes aegypti from three populations in California. Nucleotide diversity (π) across the genome in non-overlapping 100 kb windows for GC2, GC3, and Central populations. Each panel represents a chromosome, with lines and points colored by population.

Supplementary Figure 6. Boxplot of nucleotide diversity values calculated for non-overlapping 100,000 bp windows without MAF threshold.

Supplementary Table 1.

Supplementary Table 2.

Supplementary Table 3.

Supplementary Table 4.

## Data Availability

The datasets generated and/or analyzed during the current study was submitted to GenBank under Project IDs PRJNA385349, PRJNA725510 and PRJNA607233, accession number for each sample can be found in Supplementary Table 1. Codes and documentation to reproduce the analysis are publicly available on GitHub: https://github/com/vectorgenetics-lab/aedes_cali.git.

## Abbreviations

AIM: Ancestry Informative Marker
GC: Genetic Cluster
HMD: Human-Mediated Dispersal
MAD: Mosquito Abatement District
PCA: Principal Component Analysis
SNP: Single Nucleotide Polymorphisms
