# Supplementary figures and images for: Genetics of range expansion and admixture of Aedes aegypti populations in California
Source: BMC Genomics. 2025 Dec 19;26:1135. doi: 10.1186/s12864-025-12443-7 (PMC12752053; doi:10.1186/s12864-025-12443-7)

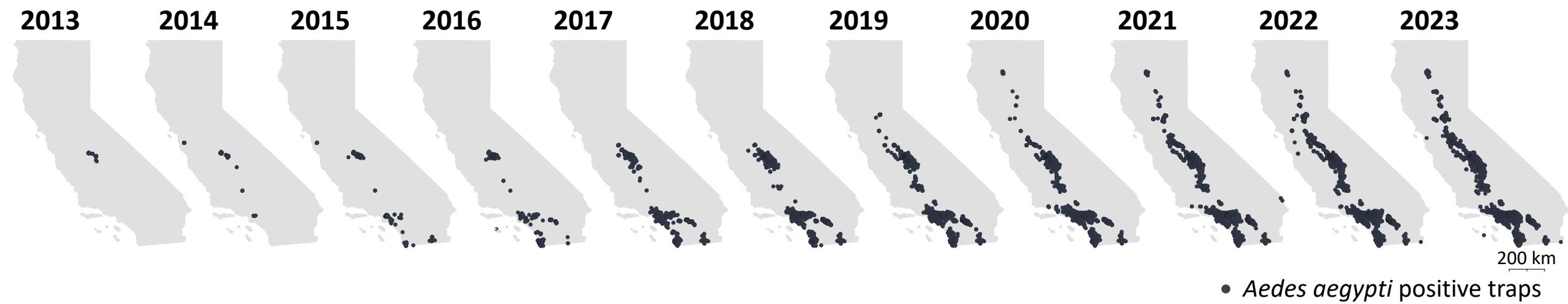

Supplement: Supplementary file 1 — Supplementary Figure 1. Range expansion of Aedes aegypti in California. Map showing the distribution of positive traps for Ae. aegypti, aggregated by year from the initial species introduction in 2013 through 2023. Data provided by the California Department of Public Health. [file 12864_2025_12443_MOESM1_ESM.pdf]

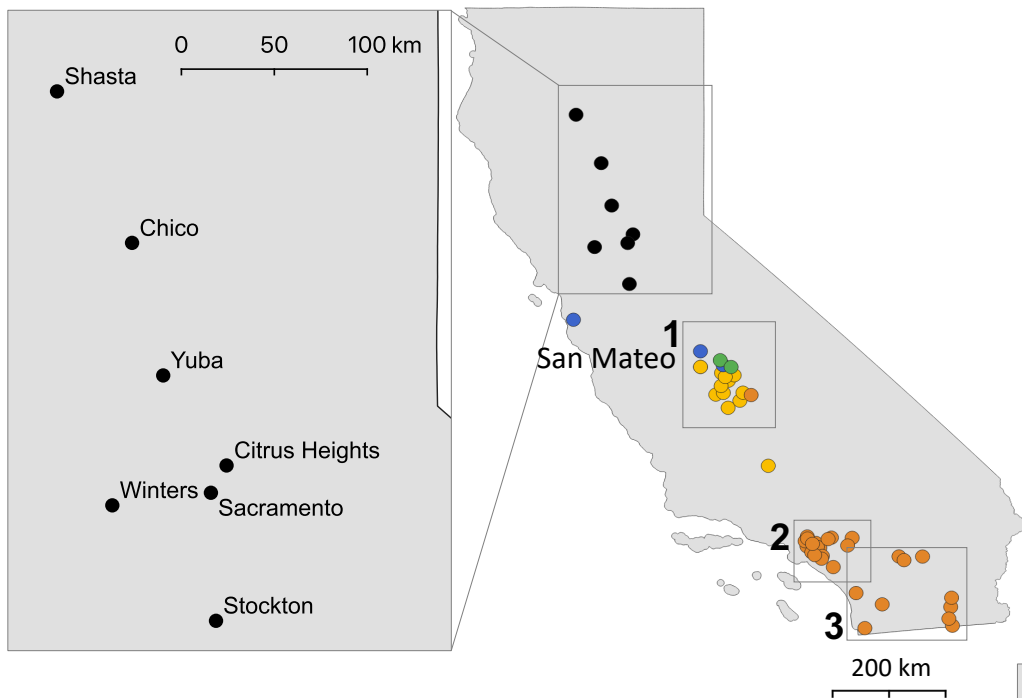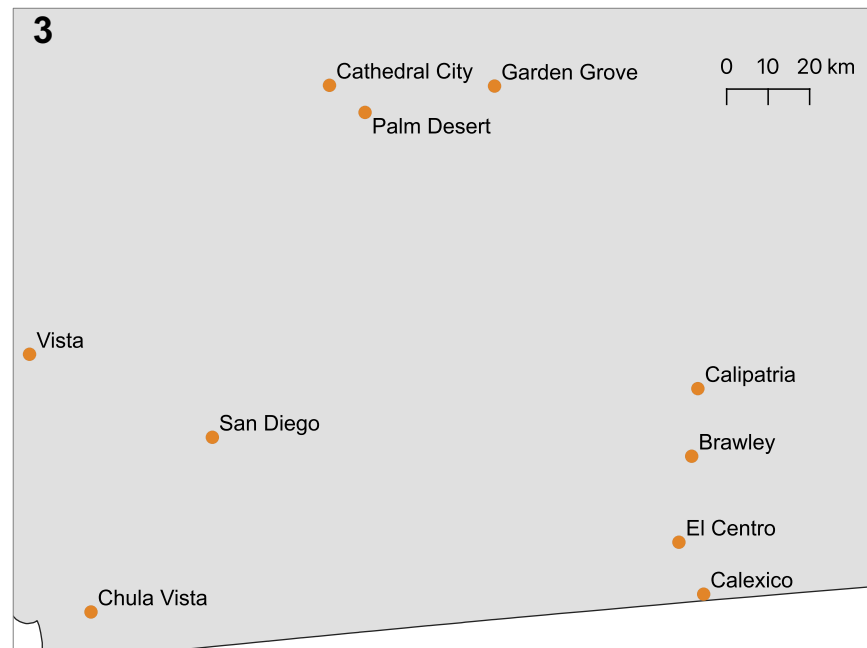

### Legend

- New Locations
- GC1
- GC2
- GC3
- Central

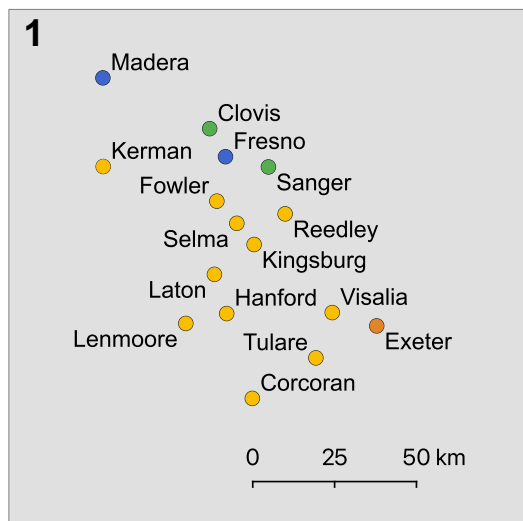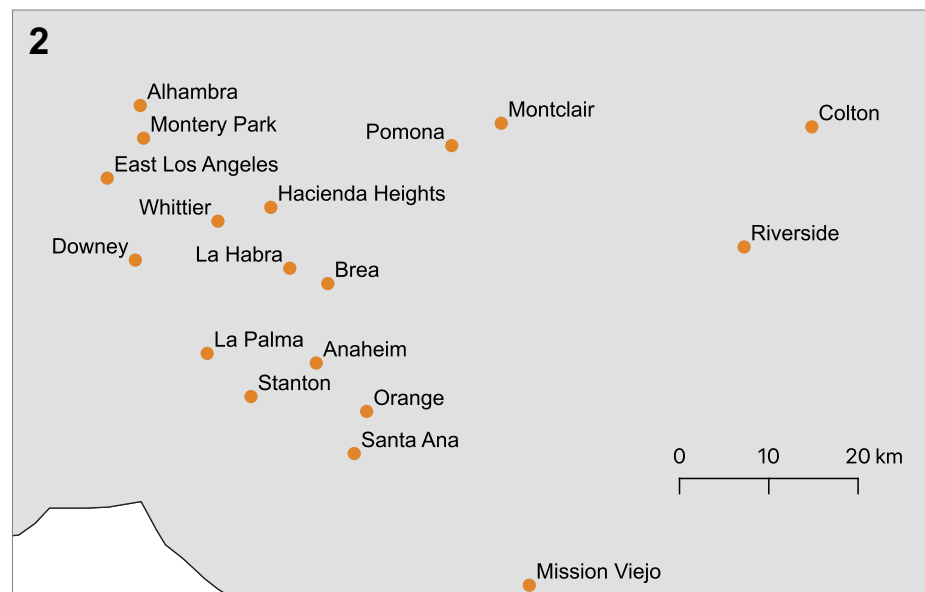

Supplement: Supplementary file 2 — Supplementary Figure 2. Additional map with all sampling location names. Sampling locations of Aedes aegypti populations in California with location names. Colors correspond to previously identified genetic clusters: GC1 (orange), GC2 (blue), and GC3 (green). Insets provide detailed views of sampling sites in the Central Valley and Northern California. [file 12864_2025_12443_MOESM2_ESM.pdf]

Cross error validation

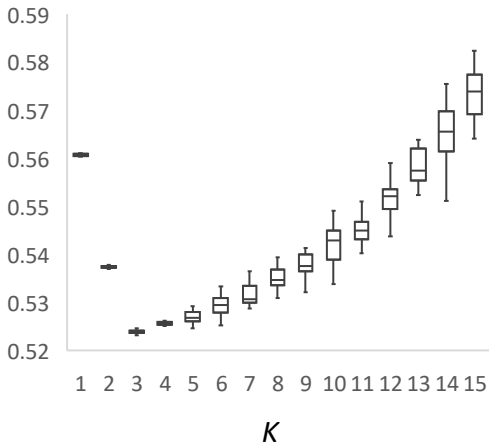

Supplement: Supplementary file 3 — Supplementary Figure 3. Cross-validation error for K from 1 to 15 of ADMIXTURE analysis; the lowest value is the best-fit number of clusters. [file 12864_2025_12443_MOESM3_ESM.pdf]

A

GC1

GC2

GC3

Central

Clovis-2019

Stockton

Citrus Heights

Sacramento

Winters

Chico

Yuba

Shasta

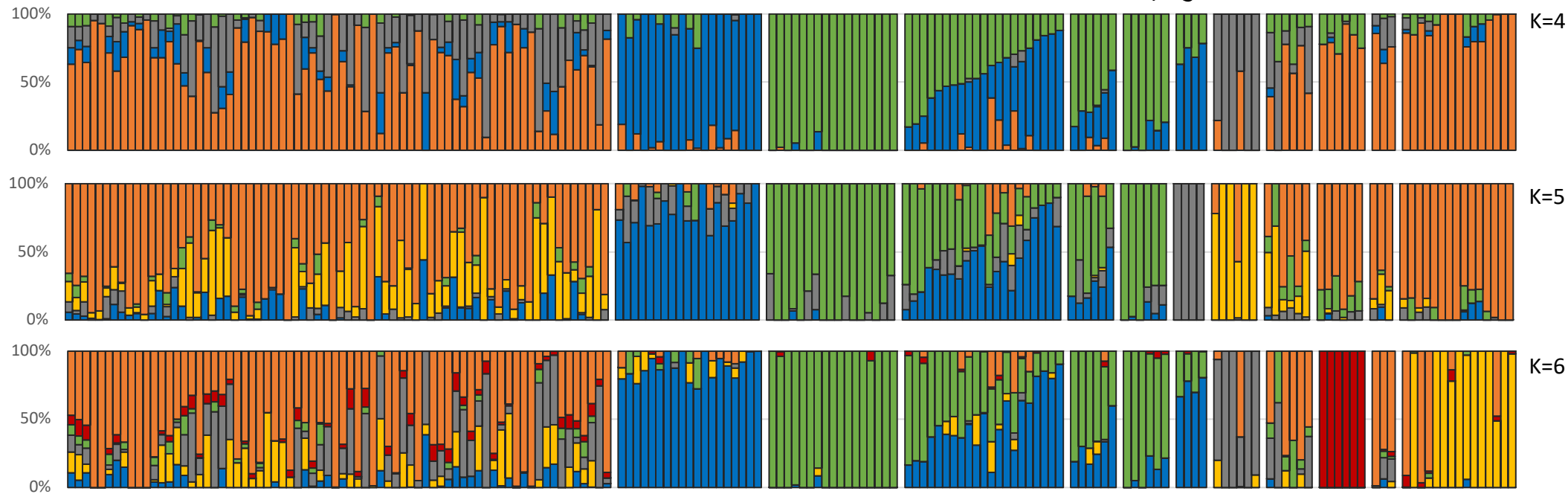

Supplement: Supplementary file 4 — Supplementary Figure 4. Bayesian analysis for ancestry estimation of Aedes aegypti populations in California. ADMIXTURE analysis of Aedes aegypti populations in California at alternative numbers of genetic clusters (K = 4, 5, and 6). Analyses were based on 50,000 SNPs across the genome. Colors represent genetic ancestry components. [file 12864_2025_12443_MOESM4_ESM.pdf]

Central

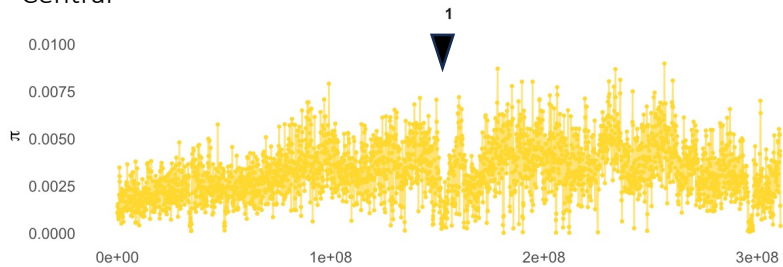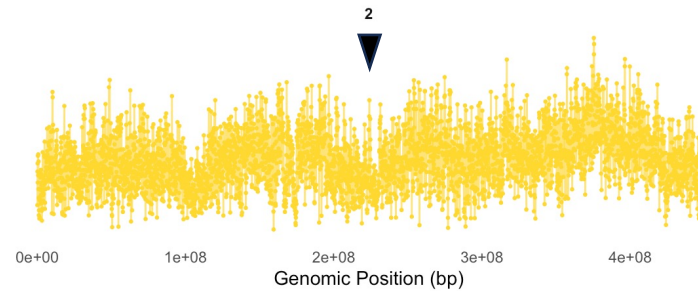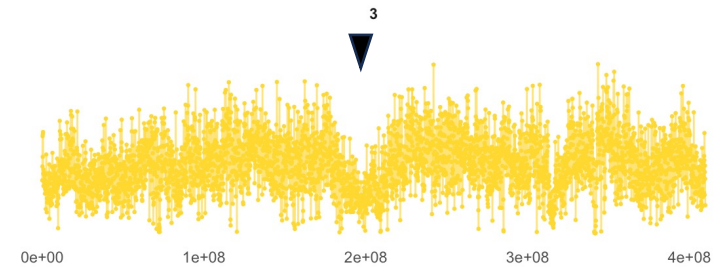

GC2

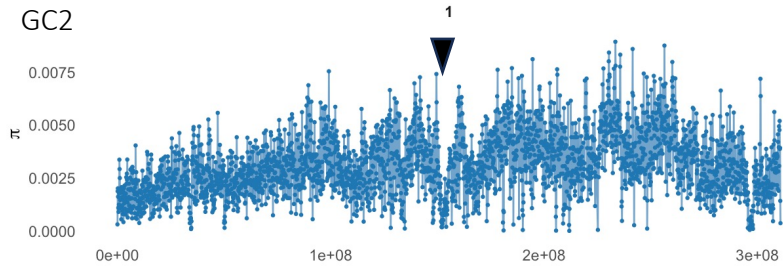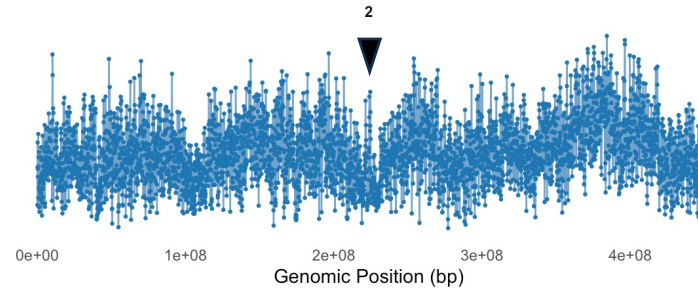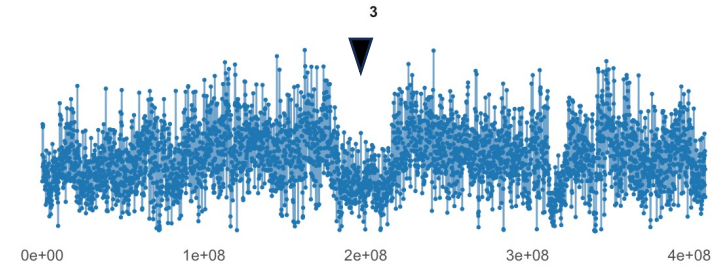

GC3

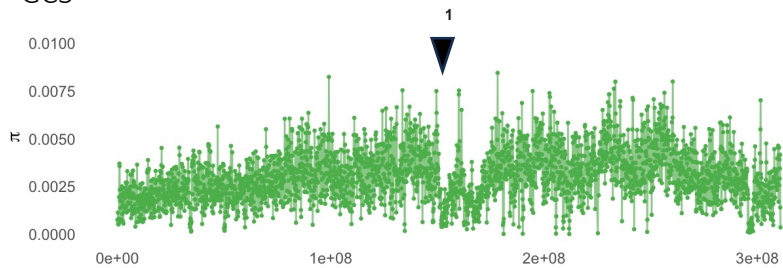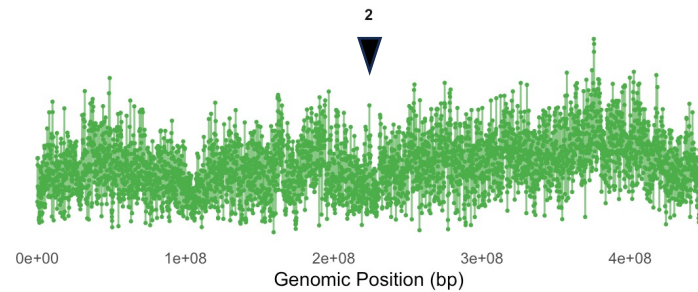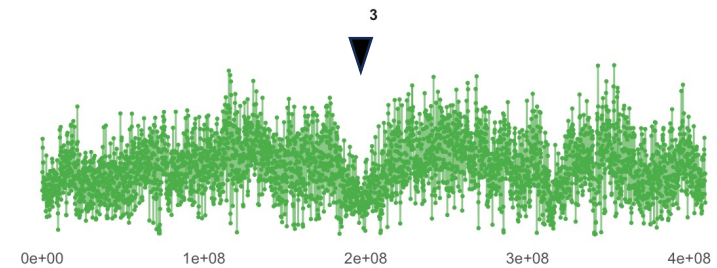

Supplement: Supplementary file 5 — Supplementary Figure 5. Nucleotide diversity across the genome of Aedes aegypti from three populations in California. Nucleotide diversity (π) across the genome in non-overlapping 100 kb windows for GC2, GC3, and Central populations. Each panel represents a chromosome, with lines and points colored by population. [file 12864_2025_12443_MOESM5_ESM.pdf]

Nucleotide diversity  $\pi$   
 $\times 10^2$

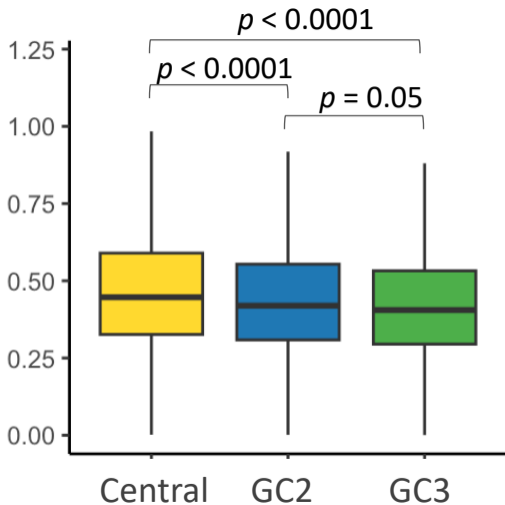

Supplement: Supplementary file 6 — Supplementary Figure 6. Boxplot of nucleotide diversity values calculated for non-overlapping 100,000 bp windows without MAF threshold. [file 12864_2025_12443_MOESM6_ESM.pdf]
